# Listing criteria for heart transplantation in the Netherlands

**DOI:** 10.1007/s12471-021-01627-x

**Published:** 2021-09-15

**Authors:** N. de Jonge, K. Damman, F. Z. Ramjankhan, N. P. van der Kaaij, S. A. J. van den Broek, M. E. Erasmus, M. Kuijpers, O. Manintveld, J. A. Bekkers, A. C. Constantinescu, J. J. Brugts, M. I. F. Oerlemans, L. W. van Laake, K. Caliskan

**Affiliations:** 1 Department of Cardiology, University Medical Centre Utrecht, Utrecht, The Netherlands; 2 Department of Cardiology, University Medical Centre Groningen, Groningen, The Netherlands; 3 Department of Cardiothoracic Surgery, University Medical Centre Utrecht, Utrecht, The Netherlands; 4 Department of Cardiothoracic Surgery, University Medical Centre Groningen, Groningen, The Netherlands; 5 Department of Cardiology, Erasmus Medical Centre Rotterdam, Rotterdam, The Netherlands; 6 Department of Cardiothoracic Surgery, Erasmus Medical Centre Rotterdam, Rotterdam, The Netherlands

**Keywords:** Heart transplantation, Advanced heart failure, Mechanical circulatory support

## Abstract

The updated listing criteria for heart transplantation are presented on behalf of the three heart transplant centres in the Netherlands. Given the shortage of donor hearts, selection of those patients who may expect to have the greatest benefit from a scarce societal resource in terms of life expectancy and quality of life is inevitable. The indication for heart transplantation includes end-stage heart disease not remediable by more conservative measures, accompanied by severe physical limitation while on optimal medical therapy, including ICD/CRT‑D. Assessment of this condition requires cardiopulmonary stress testing, prognostic stratification and invasive haemodynamic measurements. Timely referral to a tertiary centre is essential for an optimal outcome. Chronic mechanical circulatory support is being used more and more as an alternative to heart transplantation and to bridge the progressively longer waiting time for heart transplantation and, thus, has become an important treatment option for patients with advanced heart failure.

**Supplementary Information:**

The online version of this article (10.1007/s12471-021-01627-x) contains supplementary material, which is available to authorized users.

## Introduction

In 2008 a committee under the supervision of both the Netherlands Society of Cardiology and the Netherlands Association for Cardiothoracic Surgery (NVVC and NVT) published the first guidelines for heart transplantation in the *Netherlands Heart Journal* [1].

Here we present updated listing criteria for heart transplantation, on behalf of the three centres involved in heart transplantation in the Netherlands: the Erasmus MC Rotterdam, the University Medical Centre Groningen and the University Medical Centre Utrecht. These new Dutch listing criteria mainly follow the updated guidelines of the International Society for Heart and Lung Transplantation (ISHLT), and are adapted to the local situation where necessary [2].

## Present situation

The situation with regard to the number of heart transplantations in the Netherlands has not improved since 2008. On the contrary, the discrepancy between patients on the waiting list for heart transplantation and the number of donor hearts available has increased further, resulting in increasing waiting times.

**Fig. 1:**
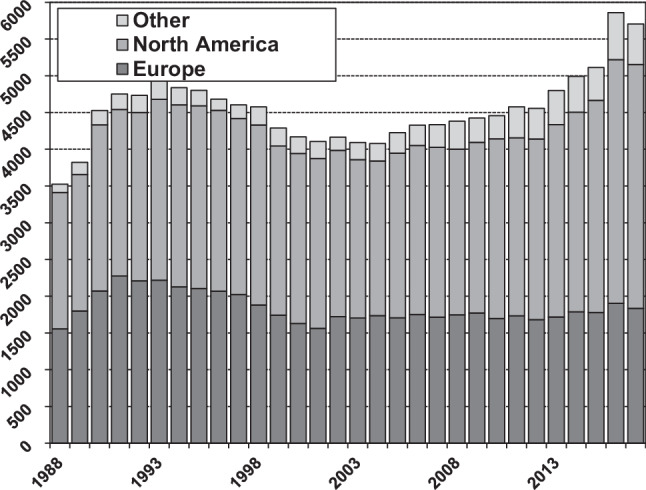
Number of heart transplantations per year according to the International Society for Heart and Lung Transplantation [3]

**Fig. 2:**
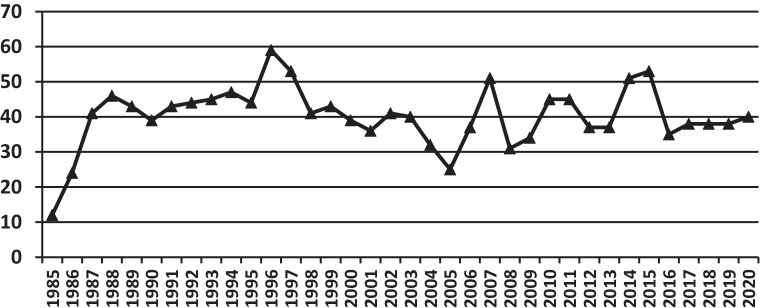
Number of heart transplantations per year in the Netherlands

Fig. 1 shows the annual number of heart transplantations worldwide, according to the ISHLT [3] and Fig. 2 shows the total number in the Netherlands. From these figures it is evident that the number of heart transplantations in Europe is more or less stable, and especially low in the Netherlands. However, the incidence and prevalence of heart failure, in general, is increasing, leading to more patients with advanced heart failure who would potentially qualify for heart transplantation. Generally, in recent years the three transplant centres together have performed 40–50 heart transplants/year, while there are ±120–140 patients on the waiting list, meaning that the mean waiting time is already approaching 3 years. It also has to be realised that the limited number of heart transplantations in Europe and especially in the Netherlands can only be performed by using older donor hearts, as can be seen in Fig. S1 (Electronic Supplementary Material) [3]. In the USA, the median donor age is still around 28 years, whereas in the Netherlands it is 47 years, with extreme cases up to 67 years of age—a very important and significant difference. This increase in donor age is mainly due to a shift in the cause of death of the donors, from younger trauma victims to elderly patients dying from cerebrovascular disease. Therefore, in general more Dutch donors suffer from pre-existing cardiovascular disease than donors in the USA. This will affect the outcome after heart transplantation, as donor age is not only a continuous risk factor for the incidence of early graft failure after transplantation, potentially leading to the death of the recipient, but it also results in more coronary artery disease late after transplantation (cardiac allograft vasculopathy) [4].

Survival after heart transplantation is good, as can be seen in Fig. S2 (Electronic Supplementary Material) and as reported by the ISHLT [3], especially considering the poor prognosis of patients with end-stage heart failure without heart transplantation.

Given the scarcity of suitable donor hearts, there is presently no room for substantial broadening of the indications for heart transplantation. This would only result in an even bigger discrepancy between the number of patients on the waiting list and the number of heart transplantations performed, with an accompanying further increase in waiting time. Therefore, careful selection of potential candidates for heart transplantation is still mandatory.

Because of the long waiting time, even for patients with acute progressive heart failure who are in urgent need of a transplant, the use of mechanical circulatory support (MCS) with a left ventricular assist device (LVAD) as a bridge to heart transplantation is growing substantially. Presently, according to the ISHLT registry, already 50% of heart transplants are performed using an LVAD as a bridge to transplantation [3].

MCS is becoming more and more important in the treatment of advanced heart failure and the mid-term outcome with regard to survival and functional recovery is approaching that of heart transplantation [5–8], although it is a very laborious and expensive therapy [9, 10]. In the near future MCS will be used more and more as an alternative to transplantation, and this will certainly have a huge impact on the selection of transplant candidates.

## Criteria for acceptance on the transplant waiting list

### End-stage heart disease not remediable by more conservative measures

In the light of the foregoing, selection of those patients who may expect to have the greatest benefit from a scarce societal resource in terms of both life expectancy and quality of life is inevitable (Tab. 1).

**Table 1:** Indication and contraindications for heart transplantation

*Indication for heart transplantation:*
– End-stage heart disease not remediable by more conservative measures
*Contraindications:*
– Irreversible pulmonary hypertension/elevated pulmonary vascular resistance
– Active systemic infection
– Active malignancy or history of malignancy with high probability of recurrence
– Inability to comply with complex medical regimen
– Severe central, peripheral or cerebrovascular disease
– Irreversible dysfunction of another organ, including diseases that may limit prognosis after heart transplantation

Patients who should be considered for heart transplantation are mainly those with severe symptoms of heart failure, and in rare cases those with intractable angina or malignant rhythm disturbances, for whom there is no alternative form of treatment available and whose prognosis is poor. In daily practice this means patients with severe, symptomatic, end-stage heart failure (New York Heart Association (NYHA) class IIIB to IV) despite (evidence-based) optimal medical and device therapy as apparent from the following:

- Maximum tolerated doses of renin-angiotensin-aldosterone system inhibitors, beta-blockers and mineralocorticoid receptor antagonists.
- CRT-P/D has been considered and/or implanted in accordance with guidelines.
- Revascularisation, rehabilitation and other interventions to improve cardiac status and quality of life of patients have been considered and/or performed.
- *V*O_2max_ ≤ 12 ml/min per kilogram on beta-blockers, ≤ 14 ml/min per kilogram in patients not on beta-blockers, or *V*O_2max_ < 50% of predicted *V*O_2_ in younger patients and women.

Strong motivation and a request by the patient to receive a heart transplant are mandatory.

The selection of patients is based on three items, which have to be combined and put into context with the rest of the data. These diagnostic criteria are: cardiopulmonary stress testing, prognostic stratification and diagnostic right-heart catheterisation [2].

### Cardiopulmonary stress testing

A maximal cardiopulmonary exercise test is defined as achievement of an anaerobic threshold on optimal pharmacological therapy with a respiratory quotient ≥ 1.05. In patients on beta-blockers, a cutoff for peak *V*O_2_ ≤ 12 ml/min per kilogram should be used to guide listing. In patients intolerant to beta-blockers, a cutoff ≤ 14 ml/min per kilogram should be used. Especially in younger patients and women, ≤ 50% of predicted *V*O_2_ can be used as an additional criterion. In obese patients, expressing peak *V*O_2_ as ml/min should be considered, to prevent falsely low values when using ml/min per kilogram. Patients should not be listed solely on the criterion of a peak *V*O_2_ measurement [2].

### Prognostic stratification

Assessment of prognosis is important in advanced heart failure to plan treatment and timely referral to a transplant centre, but can be difficult in the individual patient. Risk markers and prognostic scores are discussed extensively elsewhere [11]. Heart failure survival scores may be used together with cardiopulmonary exercise testing to guide listing for heart transplantation for ambulatory patients.

A Seattle Heart Failure Model estimated 1‑year survival of < 80% or a Heart Failure Survival Score in the high/medium risk range should be considered as reasonable cutoff points for listing. These scores are, however, not comprehensive and may overestimate survival in younger cardiomyopathy patients. Moreover, they do not incorporate haemodynamic data and cardiopulmonary exercise results.

Patients should not be listed solely on the basis of prognostic scores for heart failure survival [11–15].

### Diagnostic right-heart catheterisation

Right-heart catheterisation should be performed in all adult candidates in preparation for listing for heart transplantation and repeated annually (or more often in the case of severe pulmonary hypertension) until transplantation. Often these diagnostic catheterisations are performed in the transplant centre, after optimal medical therapy. The test is performed to assess the severity of heart failure, to support optimisation of treatment and to determine the pulmonary vascular resistance (PVR). A higher PVR correlates with worse outcome after heart transplantation [16, 17].

A vasodilator challenge should be performed when the pulmonary artery systolic pressure is ≥ 50 mm Hg and either the transpulmonary gradient (TPG = mean pulmonary arterial pressure—pulmonary capillary wedge pressure) is ≥ 15 mm Hg or the PVR is > 3 Wood units (> 240 dynes · s · cm^−5^), while maintaining a systolic arterial pressure > 85 mm Hg. Drugs used for this acute challenge are prostacyclin i.v. and nitroglycerin i.v. Other drugs, such as nitric oxide, diuretics, inotropes and vasoactive agents can be used in hospitalised patients to improve haemodynamics.

## The implications of comorbidities

Evaluation of comorbidities is important, as they may negatively affect the outcome after heart transplantation and thus have to be regarded as absolute or relative contraindications.

### Irreversible pulmonary hypertension/elevated PVR

A severely increased risk of right heart failure and mortality after heart transplantation is thought to be present [2]:

- When the PVR is > 5 Wood units (> 400 dynes · s · cm^−5^), or the PVR index is > 6 Wood units · m^2^ (in children), or the TPG exceeds 16–20 mm Hg.
- If the systolic pulmonary artery pressure exceeds 60 mm Hg in conjunction with any 1 of the 3 above-mentioned variables.
- If the PVR can be reduced to < 2.5 Wood units with a vasodilator, only at the cost of a fall in arterial systolic blood pressure < 85 mm Hg.

LVADs have been successfully used in patients with refractory elevations in PVR [18, 19]. After LVAD implantation, haemodynamics should be re-evaluated after 3–6 months, before listing for heart transplantation.

### Active systemic infection

An active systemic infection at the time of heart transplantation, when recipients are treated with high doses of immunosuppressive drugs, is still seen as an important contraindication, at least temporarily.

Persistent infections, such as human immunodeficiency virus (HIV), hepatitis B and C should be carefully analysed on an individual basis.

#### HIV

There are scarce data on organ transplantation and MCS in selected HIV patients. In these selected patients short-term survival was similar to that of the general heart transplantation population, but data on long-term outcome are lacking [20–24]. Also after LVAD implantation, short-term survival of selected HIV patients was similar to that of the general LVAD population. Only highly selected candidates may be considered if they are clinically stable and compliant to antiretroviral therapy for a long time, have undetectable HIV-RNA and CD4 counts > 200 cells/µl and have no active or prior opportunistic infection. Patients with a previous CNS lymphoma or visceral Kaposi sarcoma should not be considered [2].

The decision to accept potential candidates for heart transplantation with complex comorbidities should always consider the increasing shortage of donor hearts in general. Furthermore, it has to be realised that the management of antiretroviral therapy in combination with immunosuppressive therapy is very challenging due to substantial pharmacological interactions [22, 25] and will often hinder transplantation as a feasible solution.

#### Hepatitis B

Patients with a resolved hepatitis B infection may be considered candidates for heart transplantation, but require full serological and viral load testing at screening and every 3 months while listed as well as at the time of transplantation. In all patients with a chronic hepatitis B infection, liver biopsy should be performed to exclude severe disease. Cirrhosis, portal hypertension or hepatocellular carcinoma are contraindications to heart transplantation. Clearly, acute hepatitis B is also a contraindication [2].

#### Hepatitis C

Patients with resolved or prior inactive hepatitis C infection may be considered candidates for heart transplantation, but require full serological and viral load testing at screening and every 3 months while listed as well as at the time of transplantation. In patients with chronic hepatitis C infection, hepatitis C virus genotyping and a liver biopsy are required. Cirrhosis, portal hypertension or hepatocellular carcinoma are contraindications to heart transplantation [2].

Extensive analysis of hepatitis B and hepatitis C candidates by an experienced hepatologist is always indicated.

### Active malignancy or history of malignancy with probability of recurrence

An active neoplasm from origins other than superficial skin (basal cell carcinoma and squamous cell carcinoma) is an absolute contraindication to heart transplantation due to the limited survival rates [2]. However, patients with a history of malignancy can be considered for heart transplantation when the risk of tumour recurrence is low, preferably after a reasonable period of complete remission (at least 5 years), depending on tumour type, response to therapy and negative findings on metastatic work-up. Collaboration with oncology specialists is mandatory in all patients. LVADs can be used in these patients as a bridge to candidacy.

### Inability to comply with complex medical regimen

Compliance, the capacity to adhere to a complex lifelong regime of drug therapy, lifestyle changes and regular follow-up, is a crucial element in attaining long-term success after transplantation [2]. This includes the adequate use of all medication, because suboptimal use of immunosuppressive medication plays a major role in most acute rejections occurring more than 6 months after transplantation and it is also related to subsequent cardiac allograft vasculopathy (chronic rejection) [26], which is a major cause of mortality late after heart transplantation.

Also substance abuse (alcohol, drugs) and tobacco use have to be taken into consideration as it is thought that especially substance abuse is an important predictor of non-compliance [27]. Tobacco use continues to be the foremost avoidable cause of death in the Western world with an enormous impact on cardiovascular diseases and malignancies. Small studies have demonstrated an increased incidence of coronary allograft vasculopathy and malignancy, along with decreased survival in those patients who return to smoking after transplantation [28]. Active tobacco smoking during the previous 6 months is a risk factor for poor outcomes after transplantation and therefore considered a relative contraindication [2].

To evaluate the patient’s ability to comply with instructions, including drug therapy, a psychosocial assessment should be performed before listing for transplantation.

### Severe peripheral or cerebrovascular disease

Severe peripheral or cerebrovascular disease may contribute to both poor prognosis for survival as well as poor quality of life on a non-cardiac basis and therefore should be considered as a major comorbidity that can preclude eligibility for heart transplantation [29].

The severity of symptoms and the potential options for revascularisation may affect this decision, although it is not clear whether post-transplant risk can indeed be modified by revascularisation.

### Irreversible severe dysfunction of another organ

All comorbidities which adversely impact prognosis after transplantation should be weighed individually.

*Age* has to be seen as a continuous risk factor for outcome after heart transplantation [3]. The increased risk of older age is not so much caused by the age itself, but more by the biological age, especially in combination with frailty, cachexia and sarcopenia. Frailty includes symptoms like unintentional weight loss ≥ 5 kg within the past year, muscle loss, fatigue, slow walking speed and low levels of physical activity [30, 31].

*Chronic kidney disease* is a very important risk factor for mortality after transplantation. Irreversible renal dysfunction with a glomerular filtration rate (GFR) < 30 ml/min per 1.73 m^2^, as estimated by the creatinine clearance or estimated GFR, should be considered an absolute contraindication for heart transplantation alone [2]. In general, renal function will further deteriorate after heart transplantation, mainly as a result of the nephrotoxic immunosuppressive drugs. After heart transplantation many patients require dialysis or even a secondary kidney transplant. Although combined transplantation of a heart and a kidney from the same donor is technically feasible, it should be considered only rarely in the most appropriate individuals to maximise the limited supply of vital organs [2].

*Diabetes mellitus *with signs of end-organ damage (other than non-proliferative retinopathy alone) or persistent poor glycaemic control is a relative contraindication for transplantation [2, 32].

*Obesity*, defined as a body mass index (BMI) > 35 kg/m^2^, results in patients experiencing longer waiting times and being less likely to receive a suitable donor heart. Post-transplant morbidity and mortality are higher in such patients. Therefore, it is reasonable to strongly recommend weight loss to achieve a BMI < 35 kg/m^2^, and preferably < 32 kg/m^2^, before listing for cardiac transplantation [2, 33–35].

*Cardiac amyloidosis *is a rare disease characterised by the infiltration of misfolded proteins in several organs, such as heart, kidneys and peripheral nerves. Several types are known, of which AL (light chain) amyloidosis and TTR (transthyretin) amyloidosis may affect the heart. Evaluation and treatment should be restricted to experienced centres. AL amyloidosis is essentially a malignant haematological disease which should be treated by chemotherapy and preferably stem cell transplantation. TTR amyloidosis can be familial due to a mutation, or a result of older age (wild-type or senile). As the TTR protein is primarily produced in the liver, in mutant TTR amyloidosis liver transplantation or combined liver and heart transplantation has been performed in highly selected patients. The results of liver transplantation alone are disappointing because of ongoing wild-type TTR deposition in the heart after liver transplantation. The survival rate after combined liver and heart transplantation is better, but the numbers are low and reliable long-term follow-up is lacking [36, 37]. Recently, heart-only transplantation has also shown good outcome in carefully selected patients [38]. In wild-type TTR amyloidosis, in general older age precludes heart transplantation.

Several new drugs for the treatment of TTR amyloidosis, such as tafamidis and patisiran, recently became available, which will certainly impact future treatment [39].

## Decision making

As stated above, the indications and contraindications for heart transplantation as well as the guidelines for the acceptance of donor hearts are broadly defined. The final decision regarding acceptance is made by the heart transplant team, which has a thorough knowledge of the treatment of patients with advanced heart failure, on the one hand, and extensive experience with heart transplantation and MCS, on the other. Heart transplantation is a very limited and complex treatment modality for only a few patients. It requires a dedicated team of specialists, consisting at least of the following: cardiologists trained in advanced heart failure, heart transplantation and MCS, as well as infectious diseases and immunology; cardiothoracic surgeons with extensive experience in surgical treatments for advanced heart failure; anaesthesiologists with cardiac experience; and specialist nurses and psychologists/social workers.

To re-emphasise the point, in contrast to other complex medical therapies, heart transplantation is a form of therapy with very limited ‘resources’ and therefore requires extensive judgement to make the most optimal use of this modality. For this reason it is also important that outpatients on the waiting list for heart transplantation should be regularly re-evaluated (at least every 6 months), preferably with cardiopulmonary exercise testing and heart failure prognosis scores. If the patients have improved significantly, they should be considered for delisting [2].

In case a patient or his/her referring physician does not agree with the decision made by the transplant team of one centre, it should be possible to obtain a second opinion from one of the other centres.

## Referral

All heart failure patients should undergo regular follow-up to detect progression of symptoms and disease and to estimate their long-term prognosis. Timely referral to a tertiary centre for advanced heart failure to consider advanced therapies, such as heart transplantation and MCS, is essential [11]. Markers of advanced heart failure which may help in this referral include: requirement of i.v. inotropes, persisting NYHA class III to IV, progressive renal failure, left ventricular ejection fraction < 20%, recurrent ICD shocks, more than one hospitalisation in the previous year, persisting fluid overload or increasing diuretic requirement, low blood pressure, inability to tolerate angiotensin-converting enzyme inhibitors, angiotensin II receptor blockers, angiotensin receptor-neprilysin inhibitors or beta-blockers.

When a patient is referred to a transplant centre, extensive written and imaging data should be supplied, including a summary of the complete medical history and current data, including: cardiac and non-cardiac history; chest radiograph; results of laboratory examination; operative report in case of prior cardiac surgery; heart catheterisation data; cardiac imaging, including echocardiogram and magnetic resonance imaging; results of exercise test, if available; psychological/social information, if available.

## Allocation of donor organs

According to the Dutch law on organ donation, all organs are allocated centrally, using patient-oriented allocation according to prespecified requirements determined by the centres. Responsibility lies with the *Nederlandse Transplantatie Stichting* (NTS), which has outsourced the specific allocation to Eurotransplant (ET). Organs are allocated according to blood group, body size, medical urgency and waiting time. Final acceptance of a donor heart is the responsibility of the transplantation team, which considers all the donor data in combination with the current situation of the potential recipient. For heart donation, the upper age limit is ± 65 years. The only absolute cardiac contraindication for heart donation is the presence of significant heart disease, such as angina pectoris, myocardial infarction, prior coronary bypass surgery, moderate to severe valvular disease, cardiomyopathy or important arrhythmias. General contraindications for all donations are, for example, untreated sepsis, malignancies and infections without adequate treatment.

In the work-up of a potential heart donor, the medical history, an electrocardiogram and a transthoracic echocardiogram are essential, in addition to haemodynamic data and markers for cardiac damage, including troponin. In case left ventricular function cannot be reliably evaluated by transthoracic echocardiography, because of an insufficient acoustic window in a ventilated patient, transoesophageal echocardiography is mandatory. In haemodynamically unstable patients, a Swan-Ganz catheter should be used to optimise the filling pressures of the patient. Given the generally higher donor age in the Netherlands, coronary angiography is advised to rule out significant coronary artery disease in older donors (i.e. > 50 years) or other patients with risk factors for coronary artery disease.

With respect to the shortage of donor hearts in the Netherlands, two important initiatives have to be mentioned that hopefully will lead to an increase in the number of useable hearts. The first is the new law on organ donation, which involves active donor registration and recently came into force. The second is the recent introduction in the Netherlands of the use of donor hearts after circulatory determined death (DCD). Until recently, only hearts from donors following brainstem death (DBD) were used for transplantation. However, about half of all organ donations in the Netherlands are DCD procedures of which the kidneys, liver and lungs are used for transplantation. Recent developments in organ perfusion and retrieval techniques also allow the safe use of hearts from these donors, as was demonstrated by several centres in the UK and resulted in a substantial increase in the number of donor hearts [40].

## MCS and heart transplantation

As mentioned in the Introduction, due to the shortage of donor hearts and the progressively increasing waiting time, more and more patients are being treated by an LVAD as a bridge to transplantation. Indications and contraindications for LVAD therapy can be found in the ‘Consensus Document LVAD therapie van de Werkgroep Mechanical Circulatory Support NVT-NVVC’ [41].

Already 50–70% of the patients receiving heart transplants have had an LVAD implanted first and the expectation is that this percentage will grow even further. If the number of available donor hearts does not increase substantially, given the current promising short- and medium-term outcome after LVAD implantation, the surgical treatment of advanced heart failure will change considerably in the coming years [5].

Only patients with primarily right-sided heart failure, complex congenital heart disease or hypertrophic cardiomyopathy may undergo primary heart transplantation. All other patients will undergo long-term MCS first and only in the case of complications not amenable to LVAD replacement will heart transplantation be considered. A growing number of patients in whom an LVAD is implanted as a bridge to transplantation do already prefer not to be placed directly on the waiting list for heart transplantation [6, 9]. On the other hand, patients with advanced heart failure treated by an LVAD as an alternative to heart transplantation (so-called destination therapy) may in time qualify for heart transplantation if there is improvement in relative contraindications, such as pulmonary hypertension, renal failure and malignancy curation (bridge to decision). Thus, heart transplantation and MCS are deeply interwoven therapies and should be considered side by side in the treatment options for patients with advanced heart failure.

## Summary and conclusion

Heart transplantation is still considered to be the gold standard therapy for refractory heart failure in carefully selected patients with a high likelihood of improvement after the transplantation.

Timely referral to a transplant centre should be considered in those patients demonstrating markers of advanced heart failure, such as a requirement for i.v. inotropes, persisting NYHA class III or IV, progressive renal failure, severe left or right ventricular dysfunction, recurrent ICD shocks, more than one hospitalisation in the previous year, persisting fluid overload or increasing diuretic requirement, progressive cardiorenal syndrome and the inability to tolerate evidence-based therapy. Given the scarcity of donor hearts, careful selection of the most suitable candidates is mandatory. The growing discrepancy between potential recipients and the availability of donor hearts results in a growing number of patients who need an LVAD first, as a bridge to transplantation. New initiatives, including active donor registration and DCD heart donation, will hopefully have a positive effect on the availability of donor hearts in the Netherlands.

## Supplementary Information

Fig. S1. Median donor age by location according to the International Society for Heart and Lung Transplantation [3]

Fig. S2. Survival (%) after heart transplantation according to the International Society for Heart and Lung Transplantation [3]
