# Supplementary material for: Listing criteria for heart transplantation in the Netherlands
Source: Neth Heart J. 2021 Sep 15;29(12):611–22. doi: 10.1007/s12471-021-01627-x (PMC8630329; doi:10.1007/s12471-021-01627-x)
Supplement: Supplementary file 1 — Fig. S1. Median donor age by location according to the International Society for Heart and Lung Transplantation [3] [file 12471_2021_1627_MOESM1_ESM.docx]

Fig S1: Survival (%) after heart transplantation according to the International Society for Heart and Lung Transplantation [3]
